# Genetics-guided therapy in neuroendocrine carcinoma: response to BRAF- and MEK-inhibitors

**DOI:** 10.48101/ujms.v129.10660

**Published:** 2024-04-10

**Authors:** Lovisa Falkman, Anders Sundin, Britt Skogseid, Johan Botling, Yvette Bernardo, Göran Wallin, Liang Zhang, Staffan Welin, Ieva Lase, Kazhan Mollazadegan, Joakim Crona

**Affiliations:** a Department of Medical Sciences, Uppsala University, Uppsala, Sweden; b Department of Radiology, Uppsala University Hospital, Uppsala, Sweden; c Institute of Biomedicine, Department of Laboratory Medicine, Gothenburg University, Gothenburg, Sweden; d Department of Surgery, Örebro University Hospital, Örebro, Sweden

**Keywords:** Neuroendocrine carcinoma, neuroendocrine neoplasm, *BRAF*-mutation, BRAF-inhibitors, targeted therapy, small-molecule targeted drug

## Abstract

**Background:**

Metastatic neuroendocrine carcinoma (NEC) is associated with short survival. Other than platinum-based chemotherapy, there is no clear standard regimen. Current guidelines suggest that combination treatment with BRAF-inhibitors should be considered for patients with *BRAF* V600E-mutated NEC. However, since only eight such patients have been reported in the literature, our object was to confirm the validity of this recommendation.

**Methods:**

This was a single-center retrospective cohort study conducted at Uppsala University Hospital. The included patients 1) had a histopathologically confirmed diagnosis of NEC, 2) were diagnosed between January 1^st^, 2018 and December 31^st^, 2023, 3) had tumor tissue genetically screened by a broad next-generation sequencing (NGS) panel, and 4) showed a tumor mutation for which there is a currently available targeted therapy.

**Results:**

We screened 48 patients diagnosed with NEC between January 1^st^, 2018 and December 31^st^, 2023. Twelve had been analyzed with a broad NGS-panel, and two had a targetable mutation. Both these patients harbored a *BRAF* V600E-mutated colon-NEC and were treated with BRAF- and MEK-inhibitors dabrafenib and trametinib in second-line. At first radiological evaluation (RECIST 1.1), both patients had a reduction of tumor size, which decreased by 31 and 40%. Both had short response periods, and their overall survival was 12 and 9 months.

**Conclusions:**

*BRAF*-mutated NEC is sensitive to treatment with BRAF- and MEK-inhibitor combination. These results further support that DNA sequencing should be considered as standard of care in NECs to screen for potential treatment targets.

## Introduction

Neuroendocrine neoplasms (NENs) constitute a large and diverse group of cancers. They may arise anywhere in the body, most commonly in the gastrointestinal and broncho-pulmonary tracts (1). Previously described as *carcinoids* (2), cancer-like, they are now classified as either neuroendocrine tumor (NET, well-differentiated) or neuroendocrine carcinoma (NEC, poorly differentiated) (2–4) (Table 1). Though NETs often have an indolent presentation with a relatively slow growth rate, curable surgery is seldom possible since they tend to be metastasized at diagnosis (1).

**Table 1:** Classification of neuroendocrine neoplasms (NEN). Well-differentiated NENs are denoted neuroendocrine tumors (NET), and poorly differentiated NENs are denoted neuroendocrine carcinomas (NEC).

	Ki-67 (%)	Differentiation
**Grade I**	<3	Well-differentiated
**Grade II**	3–20	
**Grade III**	> 20	
**NEC**	> 20	Poorly differentiated

NEC, in contrast to NET, constitutes a very aggressive and poorly differentiated tumor type. While a patient with metastatic small intestinal NET experiences a median survival of 8–10 years, a patient with metastatic NEC treated with chemotherapy shows a median survival of 11–12 months (5). Digestive NECs have an annual incidence of 0.5–0.8/100 000 with a median age at diagnosis 60 years (5, 6). The only cure is surgery, made possible if the disease is localized, but more commonly, the only therapeutic option is palliative chemotherapy. Platinum-based chemotherapy with carboplatin/etoposide is generally used as first-line treatment. As second-line, fluoropyrimidines, together with irinotecan, oxaliplatin, or temozolomide, is recommended, though with limited supporting evidence (5, 7).

In the last years, genetics-guided cancer therapy has become one of the pillars of oncology. This was made possible through discoveries of oncogenic pathways and the development of specific small-molecule inhibitors. One important example is *BRAF*, an activator of the mitogen-activated protein kinase pathway (8–10) leading to cell proliferation. *BRAF* has been shown to have an activating mutation in 7% of human malignancies (11). Research has established treatment with BRAF-inhibitors together with mitogen-activated protein kinase kinase (MEK)-inhibitors (MEK being a downstream kinase in the MAPK-pathway) as the most effective and tolerable strategy. While pioneered in melanoma, it is now approved by the Food and Drug Administration (FDA) for all solid metastatic tumors harboring *BRAF* V600E-mutation where standard therapy has failed (12, 13). On the other hand, this strategy was less effective among *BRAF*-mutated adenocarcinomas of the colon (14).

Next-generation sequencing (NGS) has characterized the genetic landscape of digestive NEC. The most commonly mutated genes are *TP53* (64%), *APC* (28%), *KRAS* (22%), *BRAF* (20%), and *RB1* (14%), and copy number alterations are frequent in *MYC* (51%), *KDMA* (45%), *ARID1A* (35%), *RB1* (34%), *ESR1* (25%), and *ATM* (31%) (15). The *BRAF* V600E is (as with other cancers) the most common druggable mutation in NEC. It is mutated in 20% of NEC-cases, and in colorectal NEC, the number is even higher, 28–49% (5). While the experience of genetics-guided therapy is limited in NEC, the European Neuroendocrine Tumor Society (ENETS) 2023 guidelines (5) recommend that BRAF-inhibitor combinations should be considered as a possible treatment in case of *BRAF*-mutated tumors. However, only a limited number of patients with NEC treated with this therapy have been presented in the literature (Table 2). To validate the recommendation, we aim to describe the use of and outcome of genetics-guided therapy in NEC-patients treated at Uppsala University Hospital.

**Table 2:** Current cases describing *BRAF*-mutated NEC treated with BRAF-inhibitors. Adapted from Ricco et al. 2023 (19) PFS: progress-free survival.

Case report and reference	Primary site	Ki-67 (%)	Therapy	PFS after BRAF-inhibitor (months)	Patient status
This paper	Patient 1: Colon	35	dabrafenib + trametinib (2^nd^ line)	6	Diseased
	Patient 2: Colon	> 90	dabrafenib + trametinib (2^nd^ line)	4	Diseased
Ricco et al. (19)	Unknown	Unknown	dabrafenib + trametinib (2^nd^ line)	2	Diseased
Owaki et al. (24)	Colon	> 50	encorafenib + cetuximab + binimetinib (discontinued*) (2^nd^ line)	14	Alive
Burkart et al. (20)	Patient 1: Colon	50	dabrafenib + trametinib (2^nd^ line)	5	Unknown
	Patient 2: Colon	> 80	dabrafenib + pazopanib (discontinued*) + binimetinib (later) (3^rd^ line)	6	Diseased
Chae et al. (21)	Lung	Unknown	dabrafenib + trametinib (2^nd^ line)	At least 15	Alive
Klempner et al. (22)	Patient 1: Colon	> 60	dabrafenib + trametinib (2^nd^ line)	7	Alive
	Patient 2: Colon	> 70	vemurafenib + trametinib (2^nd^ line)	9	Alive
Imperiale et al. (23)**	Colon	Unknown	dabrafenib + trametinib (Unknown)	Unknown	Unknown
Total number of patients		10			
Median PFS excluding this paper (months)		7			

* Discontinued due to severe side effects.

** Only abstract available.

## Method

This study was approved by the Ethical Review Board in Uppsala (Dnr 2023-02359-01 and 2015-544). Patients who were alive at the time of this study provided a written-informed consent. The outline of this paper was based on Strengthening the Reporting of Observational Studies in Epidemiology (STROBE) Statement, modified to fit this particular study (16).

### Study design, setting, and participants

This single-center retrospective cohort study was conducted at Uppsala University Hospital, Uppsala, Sweden. We screened patients who had received a 1) histopathologically confirmed diagnosis of NEC 2) between January 1^st^, 2018 and December 31^st^, 2023 and identified those who 3) had molecular pathological diagnosis with a broad Next Generation Sequencing (NGS) panel (either TruSight Oncology 500 (TSO500) or Genomic Medicine Sweden 560 (GMS560)) and 4) a genetic variant for which there is a current targeted therapy (defined accordingly to OncoKB™ Therapeutic Level of Evidence level 2 (17)). Those who received targeted therapy due to said mutation were included.

### Study objective, variables, and method for outcome reporting

The objective of this study was to describe the use of genetics-guided cancer treatment of patients with NEC at our clinic and to describe the outcome of said treatment. The outcome is described as overall survival (OS) and progression-free survival (PFS) after commencing targeted therapy, as well as radiological response accordingly to Response Evaluation Criteria in Solid Tumors version 1.1 (RECIST 1.1) (18). Clinical response and toxicity were described according to the original assessment by the treating physician. All previous systemic anticancer treatment and potential side effects from targeted therapy were recorded.

## Results

**Figure 1:**
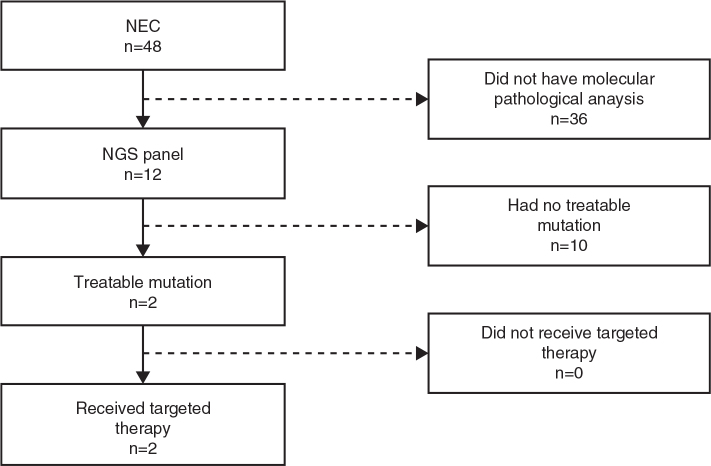
48 patients in total were referred to Uppsala Univeristy hospital after being diagnosed with neuroendocrine carcinoma (NEC) between January 1^st^, 2018 and December 31^st^, 2022. Two patients were eligible for inclusion in this study after a treatable mutation was identified using the next-generation sequencing (NGS) panel.

In total, 48 patients were identified and screened for inclusion. Tumor material for mutational analysis with a broad NGS panel was available and performed in altogether 12 subjects; four of these 12 individuals had NEC of unknown origin, three had rectal NEC, colonic primary was identified in another three, one had a tumor of the papilla Vateri, and a single patient disclosed NEC of pancreatic origin. In two of the three patients with NEC of colonic origin, NGS analyses revealed treatable mutations, and both received genetics-guided treatment (Figure 1). The following section will describe the result of NGS analyses and therapy.

### Genetics-guided therapy

Two patients had genetics-guided therapy, both presented with a colonic NEC, and DNA sequencing revealed *BRAF* V600E mutation (Figure 1).

**Figure 2:**
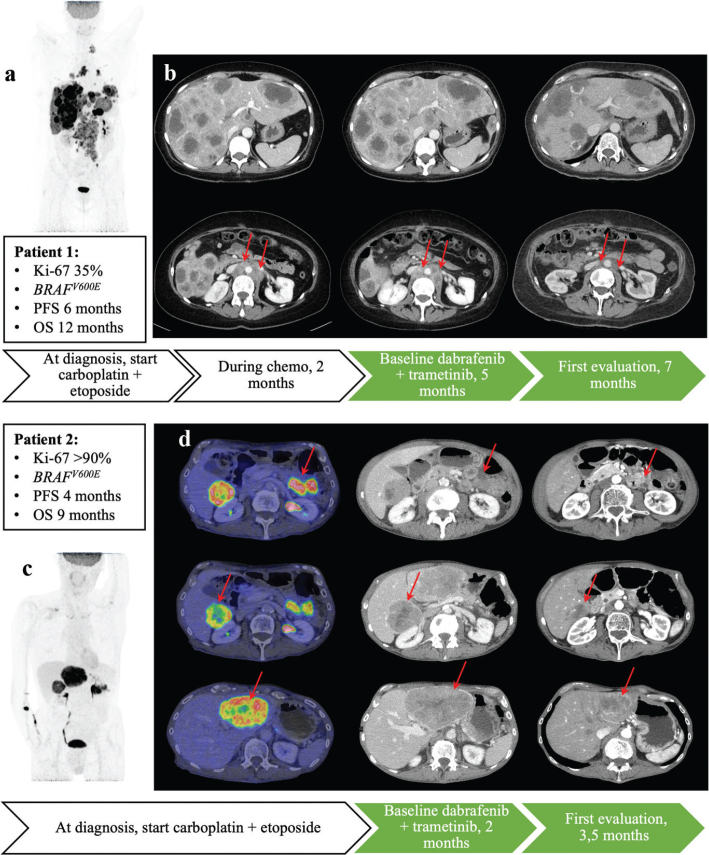
Patients having received genetics-guided therapy 2018–2023. (a) Fludeoxyglucose positron emission tomography (FDG-PET) image (Maximum Intensity Projection, MIP) of patient 1 before starting chemotherapy following surgery. (b) Patient 1’s CT images before and during treatment with dabrafenib and trametinib. First row: liver metastases. Second row: nodal metastases. (c) Fludeoxyglucose positron emission tomography (FDG-PET) image (MIP) of patient 2 before starting chemotherapy. (d) Patient 2’s FDG-PET/CT and CT images (PET/CT left, CT middle and right) before and during treatment with dabrafenib and trametinib. First row: primary tumor. Second row: right liver metastasis. Third row: left liver metastasis. PFS = progress-free survival. OS = overall survival.

The first patient was a previously healthy 53-year-old woman with metastases to abdominal and thoracic lymph nodes, lung, liver, and bone (Figure 2). She presented to the emergency department with abdominal pain, nausea, and diarrhea. Right-side hemicolectomy with resection of the primary tumor was performed due to ileus, and pathology report showed NEC with Ki-67 index 35%. In the first line, she was treated with five cycles of carboplatin/etoposide before radiological and clinical progression. NGS analysis using TSO500 revealed two pathogenic mutations, *BRAF* V600E and *TP53* R175H, tumor mutational burden (TMB) 1.6 mutation/Mb, and no microsatellite instability (MSI). BRAF- and MEK-inhibitors dabrafenib (150 mg twice daily) and trametinib (2 mg once daily) were therefore chosen as second line treatment. Figure 2a,b shows the patient’s radiological images before and during treatment. At first clinical evaluation after 1 month, the patient’s general well-being was noted to be much improved with weight-gain and less fatigue, and no relevant treatment-related side effects. After 3 months, a CT scan showed −31% in the sum of target lesion diameters according to RECIST 1.1. After 6 months, there was radiological progression with new lesions in the liver and increasing size in several other metastases. The patient reported a worsening in her general condition with pain requiring increasing opioid-dosage. She was planned for a new liver biopsy for NGS analysis to identify potential resistance mechanisms, but 1 month later, there was clinical and radiological deterioration, and the patient died shortly thereafter.

The second patient was a 71-year-old man with prostate cancer (Gleason grade 3+3, no active treatment) and multiple sclerosis who was being investigated because of weight loss. A tumor of the left colon flexure was found during coloscopy. A CT scan revealed two liver metastases, and the patient was diagnosed with colon NEC, Ki-67>90%. He was recommended palliative treatment and started first-line treatment with carboplatin/etoposide. Clinical and radiological progression was noted at the first evaluation after two cycles of treatment. At this point, a GMS560 panel had revealed the following pathogenic mutations: *BRAF* V600E, *TP53* R213*, *GNA11* F341L, *2FP36L2*, and R160fs*314. There was no MSI, and TMB was not analyzed due to limited amount of tumor cells. BRAF- and MEK-inhibitors were, therefore, selected as second line treatment, and the patient started with 150 mg dabrafenib twice daily and 2 mg trametinib daily. Figure 2c, d shows the patient’s radiological images before and during treatment. The first evaluation after 2 months showed radiological response with reduction of tumor lesions by 40%. However, the patient reported increasing fatigue, but treatment dose was left unchanged. Radiological evaluation starting 4 months after the therapy revealed tumor progression by an increase in target lesions of 23%. Dabrafenib and trametinib were discontinued, and the patient declined further treatment. He initially reported less fatigue, but there was a fast radiological progression, and the patient died shortly after.

## Discussion

This was a single-center cohort study investigating the use of genetics-guided anticancer therapy in NEC-patients at Uppsala University Hospital. Our results validate existing data that suggest potential benefit of using this concept and support current recommendations on the treatment of *BRAF*-mutated NEC using the combination of BRAF- and MEK-inhibitors.

Among the 48 patients diagnosed with NEC between 2018 and 2023 at Uppsala University Hospital, 12 patients’ tumors were analyzed by a broad NGS-panel, and two patients showed a targetable mutation. Both of these patients harbored a *BRAF* V600E-mutated colon-NEC and consequently received combination treatment with BRAF- and MEK-inhibitors dabrafenib and trametinib. Both experienced a quick and dramatic response, with a reduction of tumor target lesion sizes 31 and 40%, respectively, and, subsequently, showed fast tumor progression after 6 and 4 months, respectively.

This was a retrospective single center cohort study, which has its limitations. Already existing information in patient records was used, with the risk for information bias. The disease is rare, and patients referred to our tertiary referral center often have complicating factors resulting in selection bias. Finally, due to the low number of patients included, these results need to be interpreted with caution.

Still, we believe that our material is important as it validates recommendations in guidelines that is only supported by data from a few cases of *BRAF*-mutated NEC that responded to BRAF-inhibitor combination (19–24). Interestingly, most of these patients had colon as the primary site. Table 2 summarizes these cases and the effect of treatment with BRAF-inhibitor combination. Both patients in this study showed partial radiological response at first evaluation, and PFS of 6 and 4 months. This is slightly lower than in most previous cases, where the median PFS is 7 months. Some of the referenced patients were still responding to treatment at the time of publication but those who reported progression indicate fast deterioration. This also mirrors the need to understand the mutational progress and the importance of planning for a third-line treatment.

Despite its limitations, our report builds on current evidence that indicates that molecular pathology results in NEC may generate clinically important information in a substantial proportion of patients: two out of 12 NGS-tested patients harbored the *BRAF* V600E-mutation, and this was the only targetable mutation found. These numbers parallel previous studies. Klempner et al. (22) investigated 108 patients with colorectal high-grade NETs and found *BRAF* alterations in 9% of cases. Meanwhile, Dizdar et al. (25) report similar numbers in 71 gastro-entero-pancreatic NENs but found that in colon-NEC, the percentage of *BRAF* V600E-mutation is even higher, 46.7%. In this study, two of the three analyzed colonic NEC-patients had the *BRAF* V600E-mutation.

Another lesson from this study is the importance of acquiring tissues of progressing tumors to understand resistance mechanisms. When the NECs in this study eventually progressed, they did so very quickly, and the patients died shortly after. This shows the importance of a fast re-biopsy in order to screen for second targets and understand what genetic changes may have led to treatment resistance. For patient 1 presented in this paper, a second biopsy was planned for but never performed. Liquid biopsies, with analysis of circulating tumor DNA, could have been a viable option. Chae (21) and Klempner (22) successfully tracked *BRAF*-mutation in blood and urine of two NEC-patients. Compared to traditional tissue biopsies, liquid biopsies may provide a more nuanced picture of the disease, while also being less invasive (26–29).

It is well known that the durability of BRAF-inhibitors is limited by drug resistance (8, 30). The resistance may be intrinsic, for example, by the epidermal growth factor receptor (EGFR) signaling or by the occurrence of new mutations that activate the MAPK-pathway. Metastatic colorectal cancers, for example, show a response rate of only 5–10% to BRAF-inhibitors (8, 14, 31), which has been suggested to be attributed to a higher signaling through EGFR in response to BRAF-inhibitors (32, 33). There is one published report on a patient with colon-NEC, who was treated with dabrafenib monotherapy, but later progressed due to increase of EGFR expression (34). In contrast, basal levels of EGFR are low in melanomas (8, 34). Anti-EGFR-antibodies may enhance the effect of BRAF-inhibitors and have been reported to do so in one case of colon-MiNEN (32, 35). Trials with triplet regimes of BRAF- and MEK-inhibitors in combination with immunotherapy with immune checkpoint inhibitors have shown not only a more durable response in melanomas but also more adverse events (31). Immune checkpoint inhibitors are recommended for cutaneous melanomas following progression on BRAF- and MEK-inhibitor treatment (36).

In conclusion, the result of this study supports the ENETS’ guidelines’ recommendation to treat *BRAF*-mutated NEC with BRAF-inhibitor combinations. This should motivate genetic sequencing of NEC and treatment with BRAF-inhibitor combinations in mutated cases. However, it is important to note that the duration of response is limited, and it is important to act fast and screen for second targets once the patients’ NECs progress.
